# Preventing tuberculosis with community‐based care in an HIV‐endemic setting: a modelling analysis

**DOI:** 10.1002/jia2.26272

**Published:** 2024-06-11

**Authors:** Jennifer M. Ross, Chelsea Greene, Cara J. Broshkevitch, David W. Dowdy, Alastair van Heerden, Jesse Heitner, Darcy W. Rao, D. Allen Roberts, Adrienne E. Shapiro, Zelda B. Zabinsky, Ruanne V. Barnabas

**Affiliations:** ^1^ Division of Allergy and Infectious Diseases Department of Medicine University of Washington Seattle Washington USA; ^2^ Department of Industrial and Systems Engineering University of Washington Seattle Washington USA; ^3^ Department of Epidemiology University of North Carolina Chapel Hill North Carolina USA; ^4^ Department of Epidemiology Johns Hopkins Bloomberg School of Public Health Baltimore Maryland USA; ^5^ Centre for Community Based Research Human Sciences Research Council Pietermaritzburg South Africa; ^6^ SAMRC/Wits Developmental Pathways for Health Research Unit University of the Witwatersrand Johannesburg South Africa; ^7^ Division of Infectious Diseases Massachusetts General Hospital Boston Massachusetts USA; ^8^ Bill & Melinda Gates Foundation Seattle Washington USA; ^9^ Department of Epidemiology University of Washington Seattle Washington USA; ^10^ Department of Global Health University of Washington Seattle Washington USA; ^11^ Harvard Medical School Boston Massachusetts USA

**Keywords:** cost‐effectiveness, differentiated care, gender, HIV epidemiology, modelling, TB

## Abstract

**Introduction:**

Antiretroviral therapy (ART) and tuberculosis preventive treatment (TPT) both prevent tuberculosis (TB) disease and deaths among people living with HIV. Differentiated care models, including community‐based care, can increase the uptake of ART and TPT to prevent TB in settings with a high burden of HIV‐associated TB, particularly among men.

**Methods:**

We developed a gender‐stratified dynamic model of TB and HIV transmission and disease progression among 100,000 adults ages 15−59 in KwaZulu‐Natal, South Africa. We drew model parameters from a community‐based ART initiation and resupply trial in sub‐Saharan Africa (Delivery Optimization for Antiretroviral Therapy, DO ART) and other scientific literature. We simulated the impacts of community‐based ART and TPT care programmes during 2018−2027, assuming that community‐based ART and TPT care were scaled up to similar levels as in the DO ART trial (i.e. ART coverage increasing from 49% to 82% among men and from 69% to 83% among women) and sustained for 10 years. We projected the number of TB cases, deaths and disability‐adjusted life years (DALYs) averted relative to standard, clinic‐based care. We calculated programme costs and incremental cost‐effectiveness ratios from the provider perspective.

**Results:**

If community‐based ART care could be implemented with similar effectiveness to the DO ART trial, increased ART coverage could reduce TB incidence by 27.0% (range 21.3%−34.1%) and TB mortality by 34.6% (range 24.8%–42.2%) after 10 years. Increasing both ART and TPT uptake through community‐based ART with TPT care could reduce TB incidence by 29.7% (range 23.9%−36.0%) and TB mortality by 36.0% (range 26.9%−43.8%). Community‐based ART with TPT care reduced gender disparities in TB mortality rates, with a projected 54 more deaths annually among men than women (range 11–103) after 10 years of community‐based care versus 109 (range 41–182) in standard care. Over 10 years, the mean cost per DALY averted by community‐based ART with TPT care was $846 USD (range $709–$1012).

**Conclusions:**

By substantially increasing coverage of ART and TPT, community‐based care for people living with HIV could reduce TB incidence and mortality in settings with high burdens of HIV‐associated TB and reduce TB gender disparities.

## 1 INTRODUCTION

Tuberculosis (TB) is the leading cause of death globally among people living with HIV (PLWH) [1]. HIV acquisition markedly increases the risk of progression to active TB disease [2]. The burden of HIV‐associated TB is particularly high in South Africa, where more than 50% of people with incident TB in 2021 also had HIV, compared to a global mean of 6.7% [1]. Despite frequent co‐occurrence, TB and HIV exhibit different gender disparity patterns in South Africa, where TB prevalence nationally is higher among men and HIV prevalence is higher among women [3, 4]. Multiple factors likely contribute to greater TB burden among men, including gender differences in accessing healthcare, gender‐specific contact patterns and higher prevalence of exposures that increase risk for TB infection and/or progression (e.g. mining, incarceration, use of alcohol, illicit substances and tobacco) [5, 6, 7]. Additionally, gender disparities in HIV outcomes include lower levels of HIV viral suppression among men than women across nearly all regions globally and in South Africa [8]. The higher detectable viral loads in men contribute in part to higher incidence among women [1].

TB preventive treatment (TPT) when given with antiretroviral therapy (ART), reduces the risk of TB by approximately one‐third [9, 10, 11]. Between 2018 and 2020, over six million PLWH accessed TPT globally, exceeding the target set at the 2018 UN High‐Level Meeting on Tuberculosis. While global TPT figures are not disaggregated by sex, some studies conducted between 2020 and 2021 in sub‐Saharan Africa found lower rates of TPT initiation and completion among men than among women [12, 13], while others did not find differences [14, 15].

Differentiated models of care tailor healthcare delivery to client needs and often extend care beyond traditional facility settings and involve an additional cadre of health workers [16]. Differentiated community‐based care models have been effective and cost‐effective interventions for HIV and TB care in sub‐Saharan Africa [17], and can improve healthcare by caring for PLWH outside of health facilities. Recently, the Delivery Optimization for Antiretroviral Therapy (DO ART) household‐randomized trial of community‐based ART initiation and treatment in South Africa and Uganda demonstrated that community‐based care increases the proportion of PLWH who achieve viral suppression, particularly among men [18]. This overcame a gender disparity in viral suppression that was observed in facility‐based care models. At South African DO ART sites, asymptomatic participants in the community‐based care group without contraindications were offered TPT starting 1 month after ART initiation, while the standard clinic group received TPT per routine clinic procedures. TPT uptake was higher among participants who received community‐based care [19]. A recent modelling analysis concluded that community‐based HIV treatment was cost‐effective in preventing death and disability due to HIV [20]. However, the impacts of increased ART and TPT uptake on incident TB and TB deaths were not quantified among trial participants, nor among the surrounding community.

In this modelling study, we aim to quantify the health impact and cost‐effectiveness of extending community‐based ART and TPT care to the population scale in a TB‐HIV high‐burden setting of KwaZulu‐Natal, South Africa, including analysis of gender disparities in health outcomes. In KwaZulu‐Natal, where approximately 70% of people who develop TB also have HIV [21], we hypothesize that reaching the ART and TPT coverage levels achieved in the DO ART trial among all PLWH would substantially reduce TB disease and deaths and reduce TB gender disparities.

## 2 METHODS

### 2.1 Study design and setting

We developed a dynamic transmission model to simulate TB and HIV outcomes in KwaZulu‐Natal, South Africa. We modelled three care delivery programmes, including standard facility‐based ART and TPT care (Programme 1), community‐based ART with the facility‐based TPT initiation rate (Programme 2) and community‐based ART with TPT care (Programme 3). Programme 2 was not implemented in DO ART, but is modelled to evaluate the independent effects of ART and TPT. We executed the model over a 10‐year intervention period to project health and economic outcomes—including TB incidence, TB mortality, disability‐adjusted life years (DALYs) and costs—for each care delivery programme.

### 2.2 Dynamic transmission model

**Figure 1:**
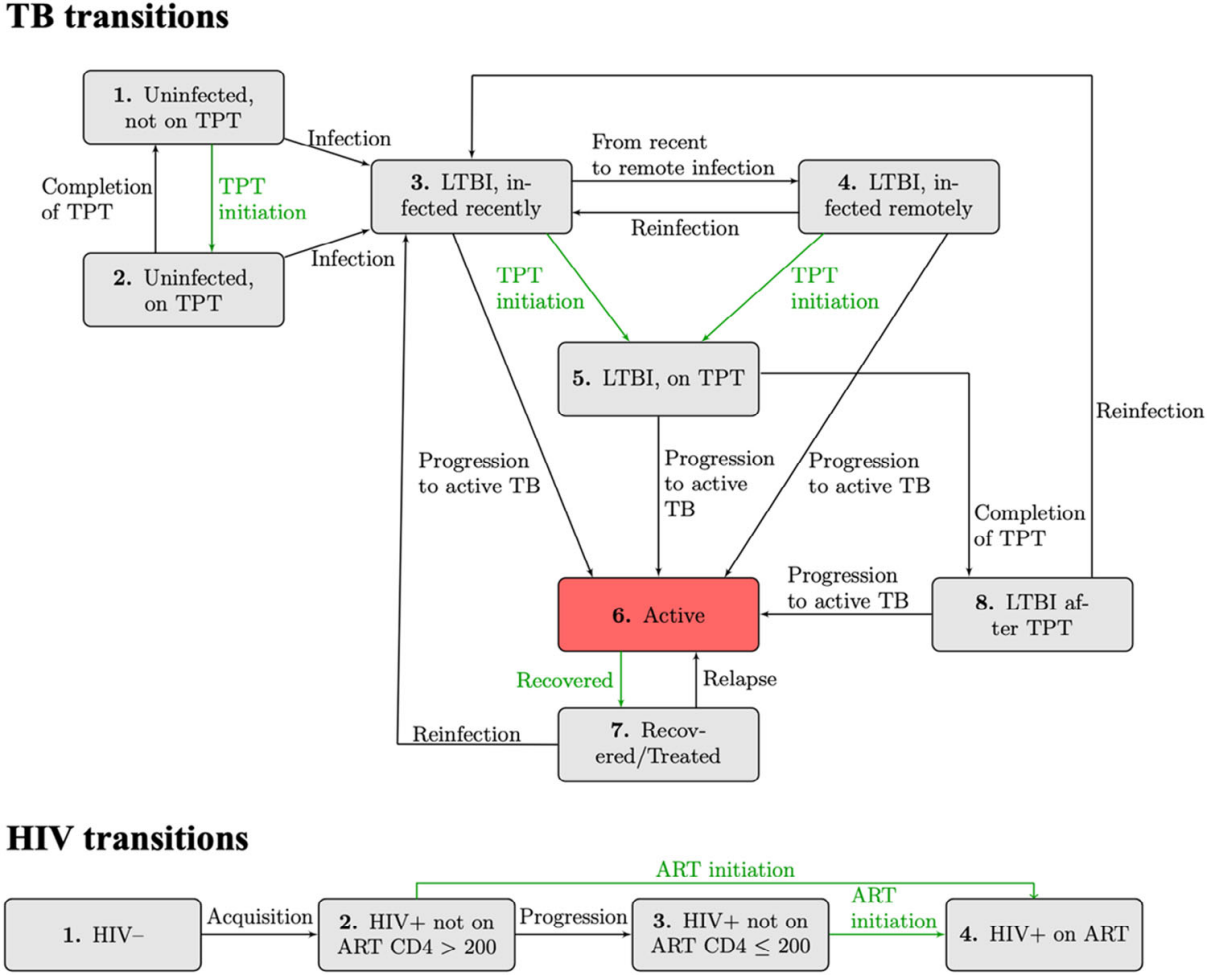
Illustration of TB and HIV dynamic transmission model. Rates of flow between each compartment are governed by differential equations, as described in the Supplementary Appendix. Although not visualized here, each TB and HIV compartment is stratified across two TB drug‐resistance categories and two genders. The latent TB infection (LTBI) compartment is distinguished by those infected within 2 years (infected recently) and more than 2 years (infected remotely). The LTBI on TPT and after TPT compartments include a mix of people who entered the compartment with recent or remote infection. The rate of TB preventive treatment (TPT) and antiretroviral therapy (ART) initiations (highlighted in green) are directly impacted by care delivery programmes (Table 2). ART coverage is used to calculate ART initiation rates so that the proportion of PLWH on ART by gender corresponds to ART coverage assumptions. The active TB compartment is highlighted in red to emphasize the compartment capturing incident TB.

The dynamic transmission model is stratified by TB stage, HIV stage, TB and HIV treatment status, TB drug resistance status and gender. We simulate a population of 100,000 persons that represent the adult population (ages 15−59) of KwaZulu‐Natal, South Africa. The population moves through the compartments at transition rates reflecting TB and HIV acquisition, disease progression, treatment and recovery from TB, death and ageing into and out of the system (Figure 1). The system of ordinary differential equations is described in the Supplementary Appendix, and is solved in R using the deSolve package in time steps of 1 month [22]. Model code is available in a public repository (https://github.com/cgreene3/epi_model_HIV_TB_KZN_SA).

The model includes TB compartments for uninfected, latent TB infection (LTBI), active TB disease and recovered/treated populations, with LTBI divided into recently infected (within 2 years), and remotely infected (more than 2 years). Those with recent infections have a higher probability of progression to active TB [23]. For simplicity, LTBI clearance [24] and recovery from active TB without treatment [25] are incorporated into rates of progression to active TB and recovery, respectively. The model considers drug‐susceptible TB (DS‐TB) and multi‐drug‐resistant TB (MDR‐TB) infections. We define MDR‐TB as resistant to isoniazid and rifampicin and assume that individuals taking TPT are not protected against acquiring an MDR‐TB infection.

The model includes HIV compartments for people living without HIV, PLWH with CD4>200, with CD4≤200 and taking ART (any CD4 count). Annual sex‐specific HIV incidence estimates for KwaZulu‐Natal, South Africa are incorporated from the Global Burden of Disease, Risk Factors, and Injuries Study 2019 (GBD 2019) [26] and the Data‐driven Recommendations for Interventions against Viral Infection model [27], as described in the Supplementary Appendix. Model parameters that differ by HIV characteristics include the relative transmissibility of TB [28, 29], risk of progression to active TB [30, 31, 32, 33, 34], duration of active TB [35] and mortality rates [30, 31, 32, 33, 34], each of which is calibrated to account for parameter uncertainty (Table 1). PLWH are eligible for TPT regardless of LTBI infection status, reflecting guidance that TPT should not be delayed among PLWH for lack of available LTBI testing [36]. We assume that only people on ART initiate TPT and those with LTBI benefit from reduced risk of progression to active TB while on TPT and afterwards [11].

**Table 1:** Dynamic transmission model input parameters

	Literature value	Accepted parameter mean	
Parameter description	[Calibration range]	[Accepted range]	Reference(s)
**Parameters that impact force of infection with TB**			
Number of effective contacts per infectious year for:			[38]
Males	14 [10.5, 17.5]	13.9 [10.5, 17.5]	
Females	14 [10.5, 17.5]	14.1 [10.5, 17.5]	
Relative transmissibility of TB (per year) for:			[28, 29]
HIV negative	1 (reference value)		
HIV positive, not on ART, CD4 > 200	0.9 [0.675, 1.125]	0.925 [0.676, 1.125]	
HIV positive, not on ART, CD4 < = 200	0.6 [0.450, 0.750]	0.601 [0.450, 0.750]	
HIV positive, on ART	0.9 [0.675, 1.125]	0.861 [0.676, 1.125]	
Fraction of new TB infections that are MDR‐TB	0.037 [0.028, 0.046]	0.037 [0.028, 0.046]	[39]
Diminished risk of acquiring latent DS‐TB infection while on TPT for those who are uninfected^a^	0.430 [0.323, 0.538]	0.431 [0.323, 0.537]	[40]
Increased risk of reinfection after developing active TB	4 [3, 5]	4 [3, 5]	[41]
Partially protective effect of LTBI against acquiring a new TB infection	0.4 [0.3, 0.5]	0.4 [0.3, 0.5]	23, 42, 43, 44
**Parameters that describe TB progression**			
Duration of TPT course	6 months (fixed value)		[18]
Duration of LTBI recently infected period	2 years (fixed value)		[23]
Annual rate of progression from:			
Recent LTBI to active TB	0.025 [0.0188, 0.0313]	0.0243 [0.0188, 0.0312]	[45, 46]
Remote LTBI to active TB	0.001 [0.0008, 0.0013]	0.0010 [0.0008, 0.0012]	[47, 48]
Relative risk of TB progression from LTBI to active TB:			[30, 31, 32, 33, 34]
HIV negative	1 (reference value)		
HIV positive, not on ART, CD4 > 200	10 [7.5, 12.5]	9.61 [7.50, 12.48]	
HIV positive, not on ART, CD4 < = 200	17 [12.75, 21.75]	16.73 [12.76, 21.24]	
HIV positive, on ART	3 [2.25, 5.25]	2.87 [2.25, 3.75]	
Annual rates of progression among PLWH and on ART (IPT efficacy against DS TB)			
LTBI on TPT to active TB	0.011 [0.0085, 0.0142]	0.011 [0.0085, 0.0142]	[49]
LTBI after TPT to active TB	0.011 [0.0085, 0.0142]	0.011 [0.0085, 0.0142]	[49]
Duration of active TB in years (if death does not occur) for:			[35]
HIV negative	2.0 [1.5, 2.5]	2.08 [1.60, 2.5]	
HIV positive, not on ART, CD4 > 200	1.5 [1.125, 1.875]	1.56 [1.20, 1.875]	
HIV positive, not on ART, CD4 < = 200	1.5 [1.125, 1.875]	1.55 [1.20, 1.875]	
HIV positive, on ART	1.5 [1.125, 1.875]	1.46 [1.20, 1.875]	
Rate of TB relapse per year	0.01 [0.008, 0.013]	0.011 [0.008, 0.012]	[50]
**Parameters that describe HIV progression**			
HIV incidence time‐varying rates^b^ for:			[26]
Males	See Supplementary Appendix		
Females	See Supplementary Appendix		
Duration from HIV acquisition to CD4 <200 in years:			[51]
Males	7.72 [5.79, 9.65]	7.79 [6.18, 9.64]	
Females	10.25 [7.69, 12.81]	10.10 [8.20, 12.81]	
**Parameters that describe mortality rates**			
Baseline mortality time‐varying rates^b^ for:			[26]
Males	See Supplementary Appendix		
Females	See Supplementary Appendix		
Increased risk of mortality for populations:			[32, 52, 53, 54, 55, 56, 57]
No active TB and HIV negative	1 (reference value)		
Active TB, HIV negative	15.5 [11.625, 19.375]	16.6 [11.63, 19.36]	
Active TB, HIV positive, not on ART, CD4 > 200	26 [19.5, 32.5]	26.2 [19.50, 32.48]	
Active TB, HIV positive, not on ART, CD4 ≤ 200	50 [37.5, 62.5]	51.2 [37.59, 62.50]	
Active TB, HIV positive, on ART	18.5 [13.875, 23.125]	18.6 [13.88, 23.12]	
No active TB, HIV positive, not on ART, CD4 > 200	8 [6, 10]	7.95 [6.00, 10.00]	
No active TB, HIV positive, not on ART, CD4 ≤ 200	26 [19.5, 32.5]	26.1 [19.50, 32.48]	
No active TB, HIV positive, on ART	1.35 [1.2, 1.5]	1.35 [1.01, 1.67]	
**Parameters that describe entries and exits due to ageing**			
Proportion of the population that enters each compartment, time‐varying	See Supplementary Appendix		[26]

*Note*: The input parameters used in the calibration include a value from the literature and range of 25% above and below the value. The accepted parameter column lists the mean and range across all 859 accepted parameter sets.

^a^ This parameter describes a reduced risk of TB infection (does not refer to active TB disease) while taking TPT. An older study is cited due to the paucity of data on this value in modern literature.

^b^ Description of time‐varying parameter calculations and mean values are in the Supplementary Appendix.

The population with active TB contributes to the force of infection for new LTBI among the susceptible population. The model allows different effective contact rates by gender, which are calibrated, but assumes homogeneous mixing in the population. The simulated population is kept constant at 100,000 by setting the total population entering the model equal to the total population exiting the model (due to ageing out or dying). We ran the dynamic transmission model from the beginning of 1940 to the end of 2027, with a warmup and calibration period from 1940 through 2017, and the intervention period from the start of 2018 through the end of 2027. We introduced HIV incidence in 1980.

We accounted for parameter uncertainty by calibrating 34 parameters using values 25% above and below the mean (Table 1). We used Latin hypercube sampling to generate 100,000 parameter sets for all 34 calibrated parameters [37]. We used each parameter set to execute the model from 1940 to 2017. Metrics from the model outputs for each parameter set were evaluated against 10 calibration target ranges in 2005 and 2017. The calibration target metrics were TB incidence and TB mortality by HIV status and sex, and HIV prevalence by sex for adults aged 15−59 in KwaZulu‐Natal, South Africa, from GBD 2019 [26]. We accepted parameter sets if all 20 metrics fell within the 95% uncertainty intervals of the calibration targets, resulting in 859 accepted parameter sets.

### 2.3 Care delivery programmes

We executed the model with the 859 accepted calibrated parameter sets for the three care delivery programmes over a 10‐year intervention period from 2018 to 2027. The programmes differ in their gender‐specific ART coverage and TPT initiation rates (Table 2). We assumed that programmes took full effect at the start of the intervention period and were maintained over 10 years. Programme 1 and Programme 3 reflect the ART coverage and TPT initiation rates observed in the facility‐based care arm and the community‐based care arm of the DO ART trial, respectively. Programme 2 reflects the levels of ART coverage observed in Programme 3, and assumes that TPT initiation occurs at the facility‐based care rate in Programme 1.

**Table 2:** Facility‐based and community‐based care programmes and parameters over the intervention period

Programme name	ART coverage %		TPT initiation % among PLWH on ART^a^	
	Male	Female	Male	Female
1. Standard facility‐based ART and TPT care	49	69	29	27
2. Community‐based ART care with standard facility‐based TPT care	82	83	17	22
3. Community‐based ART with TPT care	82	83	70	75

^a^ The TPT initiation percentages are lower in Programme 2 than in Programme 1 to adjust to the higher prevalence of ART use in Programme 2 and maintain the same number of individuals on TPT in both programmes. See the Supplementary Appendix for further information.

ART coverage estimates are based on the proportion of PLWH who achieve viral suppression under facility‐based care (Programme 1) and community‐based care (Programme 3) in the DO ART trial [18]. The proportion of PLWH on ART who achieve viral suppression and the proportion of PLWH diagnosed with HIV are based on population‐based survey data [4]. The community‐based care in Programmes 2 and 3 includes community‐wide HIV testing, so we assume that all PLWH in Programmes 2 and 3 know their status. TPT initiation rates for Programme 2 reflect the TPT initiation rates among all PLWH in Programme 1, so that the number of individuals initiating TPT in Programme 1 and Programme 2 are comparable. Details are in the Supplementary Appendix.

### 2.4 Cost model

The cost model includes costs of TB and HIV‐related care from the programme perspective (Table 3). Further details of the component costs are in the Supplementary Appendix. The annual outpatient HIV care cost with community ART delivery is estimated at $310 per person based on a micro‐costing study conducted during the DO ART trial under the “efficient at scale” scenario [18] and $249 per person for facility‐based ART delivery. Inpatient and outpatient HIV care costs exclude the cost of TB care, which is calculated separately [55]. The estimated HIV testing cost makes the assumption that programmes typically test multiple individuals for HIV before finding an eligible person to initiate ART screening. The cost of a 6‐month TPT course includes isoniazid [58], clinician time [59], the probability of developing drug‐induced liver injury from isoniazid [49] and the costs of care for drug‐induced liver injury [60]. TB care costs are estimated for standard treatment courses that reflect a higher treatment cost for MDR‐TB [61] than for DS‐TB [60] and include components for diagnostic testing, medications, laboratory testing and provider time for outpatient visits. We converted costs from the reported currency to 2018 US dollars using the consumer price index in South Africa and the United States related to the reported currency, and the currency conversion from the South African Reserve. We discounted future costs by 3%. We applied HIV‐ and TB‐related costs to the populations in relevant model compartments at each time step and summed the total HIV and TB‐related costs for each care programme under the intervention period.

**Table 3:** Cost model input parameters

Cost category	Value	Reference
Annual outpatient HIV care costs for PLWH who are^a^:		
Not on ART	135	[62]
On ART under facility‐based care (Programme 1)	249	[18]
On ART under community‐based care (Programmes 2 and 3)	310	[18]
Annual inpatient HIV care costs for PLWH who are^a^:		
Not on ART, CD4 >200	62	[63]
Not on ART, CD4≤ 200	162	[63]
On ART (regardless of facility or community‐based care)	151	[63]
Cost of HIV testing to find one person to initiate ART	24	[18, 55]
Cost of a 6‐month course of TPT with isoniazid	20	[49, 58, 59, 60]
Cost of a course of TB treatment for people with:		
Drug‐susceptible TB	259	[59]
Multidrug‐resistant TB	1889	[61]

*Note*: All values are presented as per person costs in 2018 USD and are rounded to the nearest dollar.

^a^ Inpatient and outpatient HIV care costs exclude the cost of care for TB, which is considered separately.

### 2.5 Analysis

We calculated TB‐ and HIV‐ associated DALYs as the sum of years lived with disability (YLD) and years of life lost (YLL) during the 10‐year intervention period using disability weights from GBD 2019 [64]. YLL is based on the gap between the time of death and the end of the 10‐year intervention period. We discounted costs and DALYs accrued during the 10‐year intervention period by 3%. The incremental cost‐effectiveness ratios (ICERs) per TB death, per incident TB case and per DALY averted are calculated for each care programme over the intervention period. We assessed cost‐effectiveness relative to the threshold of $590 USD per DALY averted based on the opportunity cost at the margin of the South African programme [65], as in [66]. We conducted one‐way sensitivity analyses by varying the cost parameters in the model using the mean metrics from the 859 accepted parameter sets.

## 3 RESULTS

**Figure 2:**
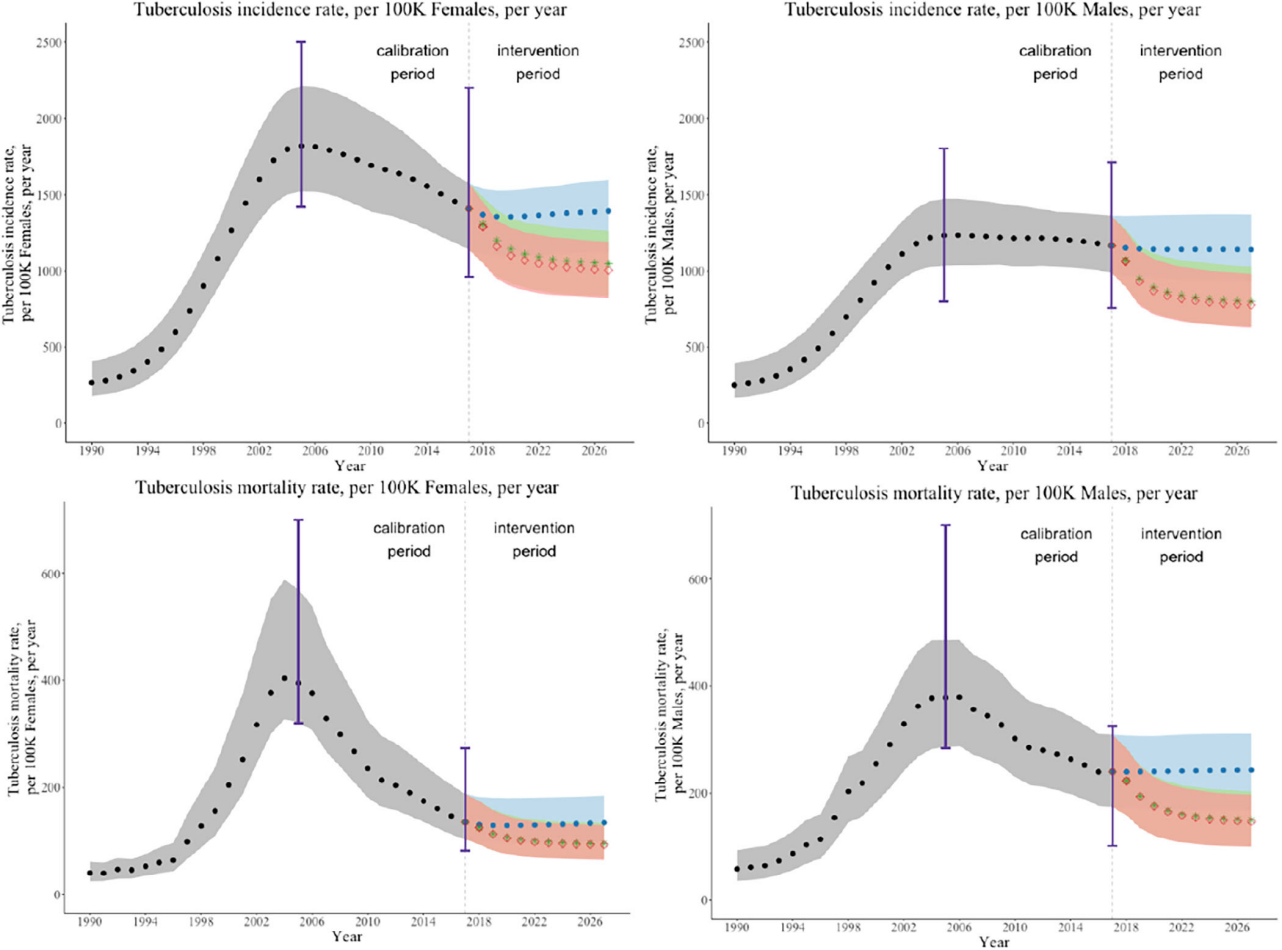
Estimated TB incidence and mortality by gender. The mean, maximum and minimum yearly TB incidence and mortality rates from 1990 to 2017 over the 859 accepted parameter sets are shown in grey (dots). The purple bars in 2005 and 2017 show the ranges of the calibration data. The mean, maximum and minimum yearly TB incidence and mortality rates during the intervention period (2018−2027) over the 859 accepted parameter sets are illustrated by care‐delivery programme. During the intervention period, Programme 1 (standard facility‐based ART and TPT care) is shown in blue (dots), Programme 2 (community‐based ART care with standard facility‐based TPT care) is shown in green (stars) and Programme 3 (community‐based ART with TPT care) is shown in red (diamonds).

Figure 2 illustrates the projected annual TB incidence and mortality rates by gender from 1990 to 2027. In 2017, the year before the start of the intervention, the estimated annual incidence of active TB disease was slightly higher for women ages 15−59 (1406 per 100,000; range 1141–1577) than for men ages 15−59 (1165 per 100,000; range 989–1362). In 2017, nearly 74.7% (1050 out of 1406) of women with incident TB were also living with HIV, compared to 62.4% (727 out of 1165) of men with incident TB also living with HIV. However, the estimated TB mortality rate in 2017 was higher for men, with an estimate of 240 (range 174–309) per 100,000 men compared with 135 (range 103–188) per 100,000 women.

Over the 10‐year intervention period, an estimated 31,009 (range 25,674–39,021) PLWH received a course of TPT in Programme 3 compared to 17,264 (range 14,156–21,255) in Programme 1 and 18,221 (range 15,305–22,370) in Programme 2 (see Supplementary Appendix for details). The estimated number needed to treat with TPT to prevent one case of active TB was 38.

The benefits of community‐based care programmes (Programmes 2 and 3) compared to standard facility‐based care (Programme 1) are most apparent at the end of the intervention period (Figure 2). TB incidence and mortality rates by gender under each programme in the last year of the intervention period are provided in Table 4. Community‐based ART with TPT care (Programme 3) reduced gender disparities in adult TB mortality compared to standard facility‐based ART and TPT care (Programme 1). TB mortality declined by 39.9% (range 32.2%–46.3%) among men ages 15−59 and 30.6% (range 25.3%−36.5%) among women ages 15−59 under Programme 3 versus Programme 1, corresponding to a smaller difference between annual TB deaths among men versus women in 2027 under Programme 3 (54, range 11−103) versus Programme 1 (109, range 41–183).

**Table 4:** Estimated TB incidence and mortality by care‐delivery programmes in the last year of the intervention period

	Programme 1	Programme 2	Programme 3
	*Standard facility‐based ART and TPT care*	*Community‐based ART care with standard facility‐based TPT care*	*Community‐based ART with TPT care*
	**Mean [Min, Max]**	**Mean [Min, Max]**	**Mean [Min, Max]**
**TB incidence**			
TB incidence rate per 100,000 men ages 15−59	1141 [977, 1301]	799 [665, 950]	775 [654, 902]
TB incidence rate per 100,000 women ages 15−59	1394 [1185, 1561]	1046 [883, 1220]	1004 [848, 1155]
**TB mortality**			
TB mortality rate per 100,000 men ages 15−59	243 [184, 300]	149 [111, 191]	146 [110, 184]
TB mortality rate per 100,000 women ages 15−59	134 [103, 174]	95 [70, 125]	93 [69, 120]

*Note*: The mean, maximum and minimum yearly TB incidence and mortality rates are estimated over the 859 accepted parameter sets.

The model projected that community‐based care programmes also had indirect health benefits for people without HIV through reduced community TB transmission. In 2027, TB incidence was 12.9% (range 8.3%–18.1%) lower among men ages 15−59 without HIV and 9.6% (range 4.7%–15.1%) lower among women ages 15−59 without HIV under Programme 3 versus Programme 1. Estimated reductions in TB mortality for people without HIV are detailed in the Supplementary Appendix.

**Figure 3:**
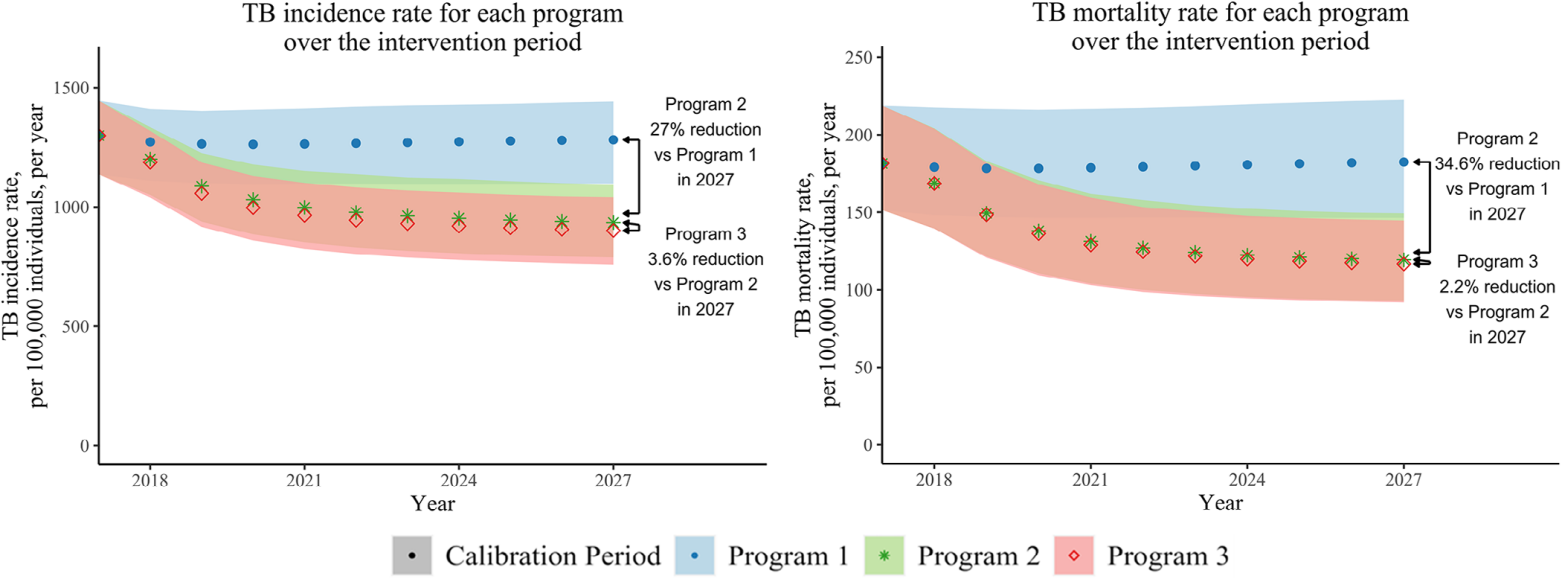
Estimated TB incidence and mortality by care‐delivery programme. The mean, maximum and minimum yearly TB incidence and mortality rates are estimated over the 859 accepted parameter sets. Programme 1 (standard facility‐based ART and TPT care) is shown in blue (dots), Programme 2 (community‐based ART care with standard facility‐based TPT care) is shown in green (stars) and Programme 3 (community‐based ART with TPT care) is shown in red (diamonds).

In Figure 3, we illustrate the population‐level impact of each programme by comparing TB incidence and mortality rates, per 100,000 individuals ages 15−59 over the intervention period. We estimated the impact of offering community‐based ART (without changing TPT uptake) by comparing outcomes in Programme 2 to Programme 1. In 2027, Programme 2 could reduce the TB incidence rate by 27.0% (range 21.3%–34.1%) and the TB mortality rate by 34.6% (range 24.8%–42.2%) compared to Programme 1 (Figure 3). We estimated the impact of offering community‐based TPT with ART by comparing outcomes in Programme 3 to Programme 2. In 2027, Programme 3 could reduce TB incidence by an additional 3.6% (range 0.2%–9.9%) and TB mortality rates by 2.2% (range 0.1%–7.6%) compared to Programme 2.

Health outcomes (DALYs, TB incident cases, TB deaths) and costs are summed over the 10‐year intervention period for each care programme (Supplementary Appendix). The incremental health gains, costs and ICERs for the outcomes of DALYs, incident TB cases and TB deaths are shown in Table 5.

**Table 5:** Incremental health gains, costs and incremental cost‐effectiveness ratios (ICERs) between community‐based and facility‐based care delivery programmes

	Programme 2 versus Programme 1 *Community‐based ART with standard facility‐based TPT care versus facility‐based ART and TPT care*	Programme 3 versus Programme 1 *Community‐based ART with TPT care versus facility‐based ART and TPT care*	Programme 3 versus Programme 2 *Community‐based ART with TPT care versus community‐based ART with facility‐based TPT care*
	Mean [Min, Max]	Mean [Min, Max]	Mean [Min, Max]
**Incremental health gains and costs (*Undiscounted*)**			
DALYs averted (thousands)	22.3 [18.1, 27]	22.5 [18.2, 27.3]	0.2 [0, 0.5]
TB incident cases averted (thousands)	2.7 [2.1, 3.5]	3.0 [2.3, 3.7]	0.3 [0, 0.9]
TB deaths averted (thousands)	0.5 [0.3, 0.7]	0.5 [0.4, 0.7]	0.0 [0.0, 0.1]
Incremental costs (millions in USD)	18.8 [16.8, 21.5]	19.0 [17.0, 21.7]	0.2 [0.1, 0.3]
**Incremental health gains and costs (*Discounted*)**			
DALYs averted (thousands)	19.7 [15.9, 23.8]	19.8 [16.0, 24.0]	0.1 [0.0, 0.4]
TB incident cases averted (thousands)	2.3 [1.8, 3.0]	2.6 [2.0, 3.2]	0.3 [0.0, 0.8]
TB deaths averted (thousands)	0.4 [0.3, 0.6]	0.4 [0.3, 0.6]	0.0 [0.0, 0.1]
Incremental costs (millions in USD)	16.6 [14.8, 18.9]	16.7 [14.9, 19.1]	0.2 [0.1, 0.3]
**Incremental cost‐effectiveness ratios (*Health gains and costs are discounted by 3%)***			
Cost per DALY averted (in USD)	843 [706, 1017]	846 [709, 1012]	1967 [244, 20,282]
Cost per incident TB case averted (in USD)	7192 [5161, 10,010]	6498 [4823, 9304]	1109 [107, 13,012]
Cost per TB death averted (in USD)	40,692 [26,116, 62,157]	39,373 [25,606, 57,020]	19,737 [1783, 470,597]

*Note*: Health outcomes and costs are summed over the 10‐year intervention period for a population of 100,000 individuals. Values are the mean, minimum and maximum values over 859 parameter sets. Discounted values are presented in 2018 values and use an annual discount rate of 3%.

**Figure 4:**
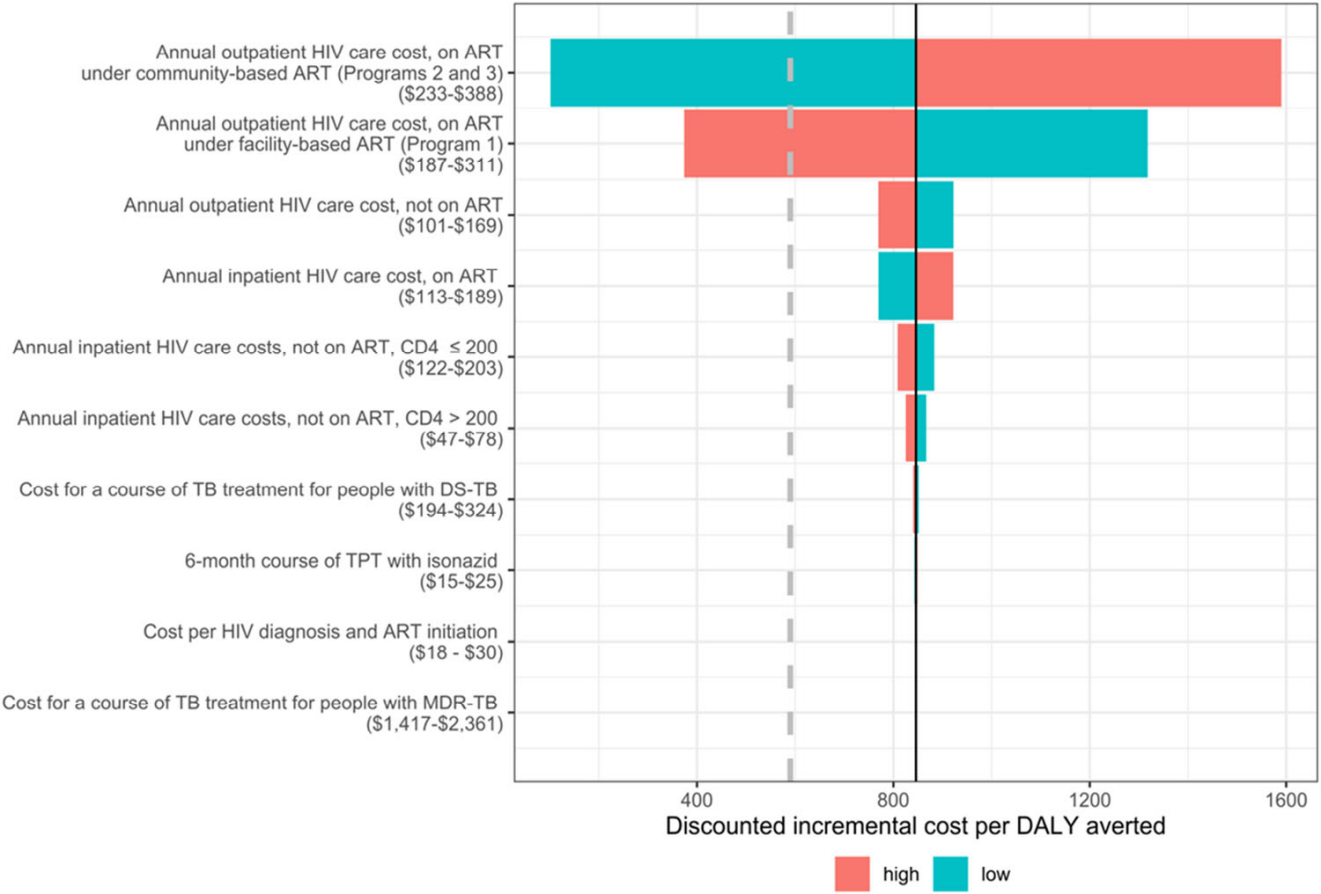
Sensitivity analysis of the discounted incremental cost per DALY averted by community‐based ART with TPT care (Programme 3) versus standard facility‐based ART and TPT care (Programme 1) over the intervention period. The solid line represents the mean discounted incremental cost per DALY averted over the 859 accepted parameter sets of $846 USD per DALY averted. The dashed vertical line represents the cost‐effectiveness threshold of $590 USD per DALY averted. The horizontal bars represent the discounted incremental cost per DALY averted at bounds of 25% above of the modelled cost parameter (high) and 25% below the modelled cost parameter (low). All costs are in 2018 USD.

The community‐based ART with TPT care programme (Programme 3) did not meet the cost‐effectiveness threshold of $590 USD per DALY averted with the input parameter costs given in Table 3. Figure 4 shows the impact of varying costs on the discounted incremental cost per DALY averted under Programme 3 versus Programme 1. The discounted incremental cost per DALY was most sensitive to the cost of outpatient HIV care. If the cost of annual outpatient care for PLWH on ART under community‐based ART programmes (Programmes 2 and 3) were to decrease from $310 to $283, or if the cost of annual outpatient care for PLWH on ART under standard facility‐based ART and TPT care (Programme 1) were to increase from $249 to $260, then the discounted incremental cost per DALY would achieve the cost‐effectiveness threshold.

## 4 DISCUSSION

Scaling up community‐based ART with TPT care to the population level in a high HIV‐TB burden setting like KwaZulu‐Natal, South Africa, could avert substantial HIV and TB morbidity and mortality, if equivalent effectiveness is maintained as demonstrated in the DO ART trial. The increase in ART coverage achieved through community‐based care reduced cumulative TB disease among people ages 15−59 by 21% over 10 years, and community‐based TPT care reduced it further by 3%. Additionally, though the interventions were only directly delivered to PLWH, the model captured the indirect benefits of reduced TB incidence disease and mortality among people without HIV. Prior modelling analyses in South Africa have also found that ART and TPT scale‐up would be projected to reduce TB incidence among people with and without HIV [67].

Gender disparities in HIV and TB outcomes in this setting are complex and are reflected in the model results. Specifically, the higher HIV prevalence among women (39%) compared to men (20%) in our model resulted in higher incidence and mortality of HIV‐associated TB among women than among men, while HIV‐negative women had lower TB incidence, prevalence and mortality than HIV‐negative men. The larger increase in ART coverage under the community‐based care programmes among men resulted in larger percent reductions in 10‐year TB‐associated mortality among men than among women compared to standard clinic‐based care. Sex and gender‐specific model parameters included effective contact rates, HIV prevalence, HIV disease progression, ART coverage and baseline (non‐HIV and non‐TB associated) mortality, though other features of TB epidemiology may also differ by gender, including care‐seeking behaviour that may impact active TB duration and case fatality ratios. A recent gender‐specific TB model in a setting with a low HIV prevalence found that interventions targeted to risk factors with a higher prevalence among men achieved the greatest projected TB incidence reductions among men, but also substantially reduced TB incidence among women and children [68].

Our cost analysis found that community‐based ART with TPT care was cost‐ineffective relative to the cost‐effectiveness threshold based on the opportunity cost at the margin of the South African programme [66]. The sensitivity analysis of cost parameters indicated that variation in the costs of outpatient HIV care for PLWH on ART substantially affected the ICER. If the annual per person costs of outpatient HIV care for PLWH on ART under community‐based care were to decrease from $310 to $283 or the annual per person costs of outpatient HIV care for PLWH on ART under facility‐based care were to increase from $249 to $260, then community‐based ART with TPT (Programme 3) could be cost‐effective. Additionally, substituting a time horizon longer than 10 years could make the intervention relatively more cost‐effective through averted YLLs, but would require additional assumptions about epidemiological trends for HIV and TB.

Strengths of this analysis include its basis in findings from the recent DO ART randomized controlled trial conducted in this setting. Additionally, the model is calibrated to sex‐ and province‐specific estimates of TB and TB‐HIV incidence and mortality at two time points that capture declines in HIV‐associated TB incidence and mortality due to increased ART coverage [5]. To reflect these dynamics, we included time‐varying parameters for HIV incidence, prevalence and ART coverage. Additionally, the model incorporates uncertainty in model parameters, as uncertainty remains in key aspects of TB epidemiology [69]. Finally, our model structure allows stratification of outcomes by gender, which is important for evaluating health programme impacts on gender equity.

This analysis also has limitations. First, we assumed that the modelled care programmes could take effect immediately and be sustained over 10 years, which may not be feasible for an already‐strained healthcare workforce and budgets. However, dramatic increases in TPT initiation and completion have been observed in some HIV high‐incidence settings through focused outreach campaigns [70]. Second, following calibration, the protective effect of ART compared to TPT was amplified, such that our model estimated a somewhat greater impact of ART and a somewhat lower impact of TPT on TB relative to our target values [11, 34]. Third, the model does not differentiate groups by age or include children under age 14 or adults older than 60 due to the complexity of calibration by age group. Fourth, we calibrated this model to GBD 2019 estimates of TB incidence and mortality by sex. While GBD 2019 estimates higher TB rates among women, owing in part to higher HIV prevalence among women, other sources (e.g. the South African National TB Prevalence Survey [3]) suggest that TB prevalence is higher among men. While this should not affect our overall estimates of TPT and ART impact, calibration to GBD data may cause our model to overestimate TB rates among women and underestimate them among men. Fifth, for simplicity, we assumed that people taking TPT would not be protected from infection with an MDR‐TB strain, though some evidence suggests that they may have partial protection from a resistant strain [71]. Finally, we assume that the duration of active TB does not change over the study period due to the lack of data to inform changes in these parameters.

## 5 CONCLUSIONS

If community‐based ART with TPT care was scaled up to achieve similar levels of ART and TPT coverage as in the DO ART trial, the dynamic transmission model predicts that TB disease would be reduced by 24% and deaths reduced by 28% over 10 years in KwaZulu‐Natal, South Africa. Differentiated models of HIV care can substantially reduce TB morbidity and mortality and reduce gender disparities in TB.

## COMPETING INTERESTS

The authors declare no competing interests.

## AUTHORS’ CONTRIBUTIONS

JMR and RVB conceptualized the study. CG formulated and programmed the model, with guidance from ZBZ and parameterization from JMR, CG, CJB, DWR, AES and DWD. AvH, DAR, AES and RVB provided critical interpretation of the DO ART trial data. All authors contributed to scenario definition and analysis planning. JMR wrote the first draft of the manuscript. CG wrote the first draft of the Supplementary Appendix. All authors contributed to the interpretation of the findings, provided critical review and revisions, and approved the final version.

## FUNDING

This work was supported by funding from the Firland Foundation. JMR is supported by the National Institute of Allergy and Infectious Diseases (K01 AI138620).

## Supporting information

Additional file 1: Appendix: The appendix file contains additional details about the study methods and additional results.

## Data Availability

Model code is available in a public repository (https://github.com/cgreene3/epi_model_HIV_TB_KZN_SA).
